# Birt–Hogg–Dubé syndrome in Chinese patients: a literature review of 120 families

**DOI:** 10.1186/s13023-021-01848-8

**Published:** 2021-05-17

**Authors:** Xiaowen Hu, Guofeng Zhang, Xianmeng Chen, Kai-Feng Xu

**Affiliations:** 1 Department of Pulmonary and Critical Care Medicine, the First Affiliated Hospital of USTC, Division of Life Sciences and Medicine, University of Science and Technology of China, Hefei, Anhui China; 2 WanNan Medical College, Wuhu, Anhui China; 3 Department of Pulmonary and Critical Care Medicine, Peking Union Medical College Hospital, Beijing, China

**Keywords:** Birt–Hogg–Dubé syndrome, *FLCN* gene, Pneumothorax, Skin lesion, Renal tumor

## Abstract

**Objective:**

To clarify the epidemiological and clinical features of Birt–Hogg–Dubé syndrome (BHDS) in Chinese patients.

**Methods:**

We identified reports on Chinese patients with BHDS by searching the China Academic Journals Database, Wanfang Chinese Database, and PubMed databases, either in Chinese or English languages published from January 1, 2008 to December 31, 2020. Studies without sufficient clinical data were excluded and cases under 18 years old were excluded.

**Results:**

Twenty papers were included and comprised 120 families with 221 cases. Most families with BHDS were reported from institutions in Beijing (66.7%) and Jiangsu Province (15.8%); 80.8% of cases were reported within the past five years. The average duration from clinical presentation to diagnosis was 9.6 years. The average age was 47.0 ± 13.9 years (range, 18–84 years) and the ratio of male to female was 1:1.6. The most common manifestations of BHDS were multiple pulmonary cysts (92.4%), spontaneous pneumothorax (71.0%), skin lesions (18.1%) and renal tumors (3.6%). Pulmonary cysts were predominantly distributed in the lower lobe on chest CT imaging. Family history of spontaneous pneumothorax was identified in 84.7% of the families and average number of pneumothoraxes was 1.8 (range, 1–6). The *FLCN* gene mutation c.1285dupC/delC in exon 11 was the most frequent mutation observed (17.4% of patients). The recurrence rate of pneumothorax after conservative treatment (including tube thoracostomy) was 29/41 (71%) while the pneumothorax recurred after surgical treatment (pulmonary bullectomy or pleurodesis) in only 4/37 (11%).

**Conclusions:**

Although BHDS has been increasingly reported in the recent years, only minority of families were reported from institutions outside of Beijing and Jiangsu Province. The dominant clinical manifestations were pulmonary cysts associated with recurrent pneumothorax, while skin lesions and renal tumors were less commonly reported. Delayed diagnosis along with suboptimal management appear to represent critical challenges for Chinese patients with BHDS.

## Background

Birt–Hogg–Dubé syndrome (BHDS, OMIM#135150) is a rare autosomal dominant inherited disorder, first described in 1977, featured by lung cysts, spontaneous pneumothorax, skin lesions (fibrofolliculomas and trichodiscomas) and renal tumors [1]. It is caused by germline mutations in the folliculin (*FLCN)* gene, which is located on chromosome 17p11.2 and encodes a 579-amino acid protein with tumor suppressor function, and interacts with the mammalian target of rapamycin(mTOR) and adenosine monophosphate-activated protein kinase (AMPK) signaling pathways [2–5]. Over 600 families with BHD have been reported worldwide in the past 40 years, mainly Caucasians in Europe and USA. To date, over 200 *FLCN* gene variants have been identified to cause BHDS according to the Leiden Open Variation Database (https://www.lovd.nl/).

Multiple bilateral pulmonary cysts are the most common manifestation in both Asian and Caucasian individuals with BHDS. Fibrofolliculomas are highly specific manifestation occurring in more than 80% of Caucasian individuals with BHDS. Renal tumors develop in up to one-third of individuals with BHDS, with an average age of onset of 50 years reported in Caucasian individuals [6]. In East Asia, Japanese authors reported over 100 families with less frequent skin and renal manifestations [7].

In 2008, the first detailed report of BHDS in Chinese patients focused on the gene mutations identified among patients manifesting spontaneous pneumothorax in Jiangsu province [8]. In 2017, Liu et al. provided a systematic genetic and radiological description of a BHDS case series in the Chinese population [9]. Until recently, data on clinical features of Chinese patients with BHD were limited to case reports or small series, mainly with pulmonary features. Extrapulmonary abnormalities, including skin and kidney manifestations, are uncommonly reported in Chinese individuals of BHDS [10]. With an increasing number of reports published in recent decades, it seemed warranted to summarize the prevalence and clinical characteristics associated with BHDS in the Chinese population in order to clarify differences between Chinese and Caucasian individuals with this disorder.

The purposes of this study are to fill this knowledge gap by exploring the epidemiology and disease characteristics of BHDS reported in China, focusing on clinical, radiological, genetic, diagnostic, and therapeutic aspects to improve recognition of this rare disease in the Chinese population.

## Material and methods

### Study design and data collection

Firstly, the following keywords “Birt–Hogg–Dubé syndrome” or “BHD” or “BHDS” [All Fields]) combined “China” or “Chinese” in the China Academic Journals Full-text Database (CAJ), Wanfang Chinese Database and PUBMED English Database were searched by Dr. Zhang in September 2020, and then updated in December 2020. This was supplemented with manual search to identify other relevant documents as conducted by two authors (Drs. Hu and Zhang). Articles were restricted to those published and available as full texts in either Chinese or English languages; conference abstracts were excluded. We included BHDS cases reported in China from January 1, 2008 to December 31, 2020; the diagnosis of BHDS was based on the criteria proposed by the European BHD consortium. BHDS could be diagnosed by fulfilling one major criterion or two minor criteria. The major criteria included: (1) at least five fibrofolliculomas or trichodiscomas (at least one histologically confirmed, of adult onset); (2) pathogenic *FLCN* germline mutation. The minor criteria included: (1) multiple lung cysts (bilateral basally located lung cysts with no other apparent cause, with or without spontaneous pneumothorax); (2) renal cancer (early onset before age 50 or multifocal or bilateral, or mixed chromophobe and oncocytic histology); (3) a first-degree relative with BHDS [11]. Patients must have been living in China (including from Taiwan, Hong Kong, or Macau) and the papers must have provided enough information about the clinical features, including medical history, imaging data, gene results and therapeutic strategy. Exclusion criteria included duplicate reports, basic science research, and clinical studies without sufficient clinical data. Cases under 18 years old were excluded from the final analysis. The study was waived by the institutional review board of the First Affiliated Hospital of University of Science and Technology of China because the data were obtained from published reported and were analyzed anonymously and retrospectively.

Clinical information including sex, age, family history, pulmonary cysts, detailed history of pneumothorax, skin lesion, renal tumor, gene testing, diagnosis, treatment, and outcome was collected.

### Statistic methods

All data was expressed in the form of means and standard deviations (${\overline{\text{x}}} \pm {\text{s}}$). SPSS 25.0 was used for data analysis and independent sample t-test was used to compare continuous variables. Chi-square test was used to compare categorical variables. Differences with a value of *P* < 0.05 was considered statistically significant.

## Results

### Demographic data

**Fig. 1:**
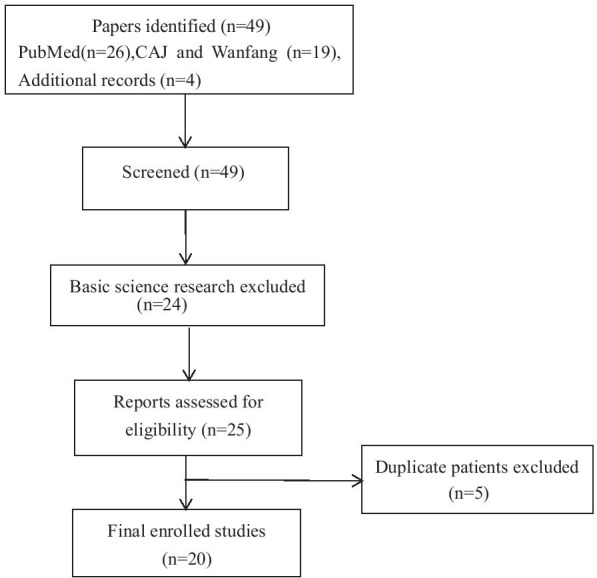
The flow diagram of this study. Abbreviations: CAJ = China Academic Journals Full-text Database (CAJ), Wanfang = Wanfang Chinese Database

**Fig. 2:**
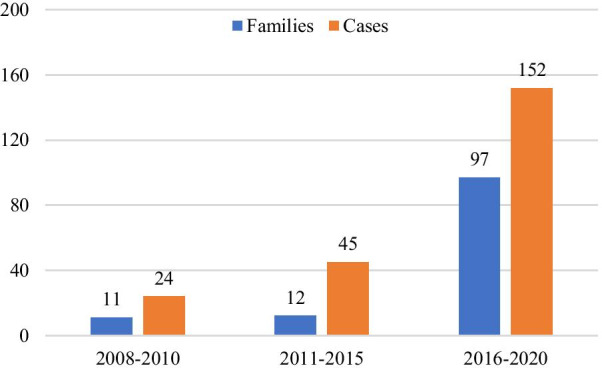
Number of families and cases of BHDS in China distributed by year of report

There were 49 BHDS-related papers in English or Chinese languages by Chinese authors between January 1, 2008 and December 31, 2020 (Fig. 1). Using the study criteria outlined above, 221 cases from 120 families with BHDS were included in the final analysis, which were reported in 20 papers (Table 1) [8–10, 12–28]. In 2008, the first 10 families with spontaneous pneumothorax and positive *FLCN* mutations in the Chinese population were described by a research team at Nanjing University [8]. There were 26 BHD families reported in 2020 and 34 in 2019,2 in 2018, 33 in 2017, 2 in 2016 (in Table 1). As shown in Fig. 2, 97 (80.8%) of 120 families were reported in the past five years. Thus, 11 and 12 BHDS families were reported in 2008–2010 and 2011–2015 periods, respectively. Furthermore, 60 (50%) families were reported in the past two years, and was equal to the number of cases reported in the preceding ten years. Although the reporting regions included 9 Chinese provinces (Table 2), most of the cases came from Beijing (80 families, 66.7%), followed by Jiangsu province (19 families, 15.8%). Among 221 patients, 38.9% were male and the ratio of male to female was 1:1.6. The average age at the time of diagnosis was 47.0 ± 13.9 years (range, 18–84 years).

**Table 1:** The features of enrolled papers of BHDS in the final study

Author	Subspecialty journals	Province	Year of publication	Number of families	Number of cases
Ren H-Z	Genetics	Jiangsu	2008	10	23
Shunyang So	Respiratory	Hong Kong	2009	1	1
GeeGwo Yang	Respiratory	Taiwan	2013	1	1
Zhichun Lin	Medicine	Beijing	2014	1	3
Zhibo Liu	Genetics	Beijing	2014	1	1
Yibing Ding	Genetics	Jiangsu	2015	9	40
Li Dong	Medicine	Tianjin	2016	2	2
JF Zhu	Medicine	Hubei	2017	1	1
Lv Liu	Genetics	Hunan	2017	2	8
Yaping Liu	Rare Diseases	Beijing	2017	27	27
Teng Li	Cancer	Beijing	2017	2	2
Shengyu Hao	Respiratory	Shandong	2017	1	1
Xiaocan Hou	Genetics	Hunan	2018	2	8
Fei Xie	Urology	Beijing	2019	1	1
Keqiang Liu	Rare Diseases	Beijing	2019	31	38
Qun Hu	Respiratory	Fujian	2019	1	1
Chunming Zheng	Medicine	Beijing	2019	1	10
Yanguo Liu	Surgery	Beijing	2020	16	37
Dandan Zong	Respiratory	Hunan	2020	1	7
Ting Guo	Medicine	Hunan	2020	9	9
TOTAL				120	221

**Table 2:** BHDS according reporting provinces in China

Province	Family (%)	Case (%)
Beijing	80 (66.7)	119 (53.9)
Jiangsu	19 (15.8)	63 (28.6)
Hunan	14 (11.8)	32 (14.6)
Tianjin	2 (1.7)	2 (0.9)
Shandong	1 (0.8)	1 (0.5)
Hubei	1 (0.8)	1 (0.5)
Fujian	1 (0.8)	1 (0.5)
Hong Kong	1 (0.8)	1 (0.5)
Taiwan	1 (0.8)	1 (0.5)
Total	120	221

### Clinical manifestations

Spontaneous pneumothorax was observed in 157 patients; the characteristics of pneumothorax was described in 78 of these patients, including the age at initial pneumothorax, number of episodes, and side of pneumothorax. The average age at first pneumothorax episode was 39.2 ± 12.7 years (range, 18 to 71). The average number of pneumothorax per patient was 1.8 ± 1.1 (range, 1 to 6). Among these, 74 and 4 patients experienced unilateral and bilateral pneumothorax, respectively. Family history of pneumothorax was identified in 84.7% patients.

Among 221 patients, 40 (18.1%) patients were found to have skin lesions when they were diagnosed as BHDS, including 6 fibrofolliculomas, 1 with sarcoma [9], 11 with papules, and 22 cases without specific descriptions. Eight patients had renal cancers (3.6%) of whom two cases of renal cell carcinoma were not described in detail. One patient was diagnosed with bilateral renal carcinomas including chromophobe cell carcinoma on the left and clear cell carcinoma on the right. The remaining 5 patients had unilateral renal carcinoma. Thyroid carcinoma was found in two patients from two different families and uterine myoma was discovered in one case from Hunan Province.

### Imaging findings

There were 195 patients (92.4%) with multiple pulmonary cystic lesions on chest CT (Table 3). The detailed locations of lung cysts were described in 75 cases; the cystic lesions were located bilaterally in 52 cases (69.3%), and most of them in the lower lung zones. Most of the cysts ranged 0.5-6 cm in diameter; the largest cystic lesion measured 8.5 cm.

**Table 3:** Comparison of characteristics among patients with BHDS between China, Japan and USA

Characteristic	China#	Japan^+^	USA^$^	*P* value
Pneumothorax (n, %)	157 (71.0%)	230 (73.7%)	79 (76.0%)	0.616
Cysts on chest CT (n, %)	195 (92.4%)	309 (99.0%)*	88 (85.0%)	< 0.01
Skin lesion (n, %)	40 (18.1%)	76 (48.7%)*	74 (71.0%)*	< 0.01
Renal tumor (n, %)	8 (3.6%)	40 (34.8%)*	34 (33.0%)*	< 0.01

^*^P <  < 0.05, #Current study; + data from Clin Genet 2016, 90: 403–412; $data from Ann Am Thorac Soc 2017,14 (5): 706–713

### *FLCN* mutations

All 221 patients were diagnosed by genetic testing and the results were reported in detail for 144 of them. The mutation in exon was found in 134 patients, including almost all exons 4–14. The most frequent mutation (17.4%, 25/144) was the single deletion, duplication of cytosine in codon 1285 of exon 11 (c.1285del, c.1285dup), follow by the mutation of c.1579_1580ins in exon 14 (10.4%, 15/144), as was reported in previous studies, and a three base pair deletion in exon 6 (c.924_926del) (9.0%,13/144) was the third most common mutation Other mutations occurred much less frequently. Mutation c.924_926del was associated with a 39% risk (5 of 13 patients) for pneumothorax, which increased to 60% for mutation c.1285dup (9 of 15 patients) and 73% for mutation c. 1579_1580ins (11 of 15 patients). However, the differences in the risk of pneumothorax associated with these three most common mutations was not statistically significant (patients must have experienced at least one pneumothorax episode) ($\chi^{2}$, 3.513; *P* value, 0.173). The mutant gene in 6 patients with fibrofolliculomas included exon 11, 9, 13; 8 renal carcinoma patients manifested mutations in exon 7, 9, 10, 11, 13, 14.

### Diagnosis

All 221 patients were confirmed to have BHDS by detection of pathogenic variant in the *FLCN* gene. Detailed information was available for 32 families that were initially misdiagnosed. Almost all initial presentations involved pneumothorax, which was often misdiagnosed as primary spontaneous pneumothorax. One case reported from Fujian Province was misdiagnosed as lymphangioleiomyomatosis (LAM) and had been on rapamycin (sirolimus) treatment for several weeks. The average duration of misdiagnosis was 9.6 ± 10.8 years (range from 0 to 36 years) prior to the eventual diagnosis of BHDS.

### Treatment

The detailed information regarding management of pneumothorax was available in 57 patients. 37 BHDS patients with pneumothorax received surgical treatment. Among them, 29 patients with pneumothorax were treated by bullectomy with pleurodesis; pneumothorax recurred in three cases. The remaining of pneumothorax were treated by pleurodesis alone and the other 5 by bullectomy. Pneumothorax recurred in one patient after pleurodesis. The recurrence rate of pneumothorax after conservative treatment (including tube thoracostomy) was 29/41 (71%) while the pneumothorax recurred after surgical treatment (pulmonary bullectomy or pleurodesis) in only 4/37 (11%). Partial nephrectomies were performed in all renal cancer patients but only one patient had a three-month follow-up after surgery. Nevertheless, the remaining patients had no following up visits until publication.

### Prognosis

The follow-up information for patients with BHDS in China were rarely described in the published reports. Among 43 patients with recurrent pneumothoraces, ipsilateral and contralateral recurrences were described in 67% and 33%, respectively. Only one patient in Beijing was followed up after open partial nephrectomy and there was no new abnormality in the kidneys at three months.

## Discussion

To the best of our knowledge, this study comprises the largest number of families of BHDS in the Chinese population. We found most BHDS families were reported from Beijing and Jiangsu Province, and over 80% of them were reported in only the past five years. In comparison to USA and Japanese BHDS cohorts, the dominant manifestations of Chinese patients were pulmonary cysts associated with recurrent pneumothorax, while skin lesions and renal tumors were less reported. Delayed diagnosis and suboptimal medical management presented common challenges for Chinese patients with BHDS.

BHDS is a rare inherited autosomal dominant disorder caused by germline mutations in the tumor suppressor gene *FLCN* that predisposes affected individuals to develop benign skin tumors (fibrofolliculomas), renal neoplasms, and pulmonary cysts with a risk of spontaneous pneumothorax. Since a research team from Nanjing University firstly explored the *FLCN* gene mutations on patients with spontaneous pneumothorax history in 2008, there has been more than 20 families with BHDS in China up to 2017 according to the BHD foundation statistics. Our study included 221 cases from 120 families that were reported between January 2008 and December 2020 in China. More than 80% of families were described in the past five years, half of them in the past two years. The increasing number of BHDS in China likely reflects the increasing awareness and recognition rather than increasing incidence of disorder. In addition, medical resources and economic levels in different regions may also lead to the regional distribution of patients with BHD syndrome. In recent decades, a fascinating progress has been achieved with respect to the diagnosis and management of rare diseases in China. A registry study on rare respiratory diseases including another diffuse cystic lung disease, LAM, has been ongoing nation-wide since 2016. Therefore, BHDS is also being increasingly diagnosed by clinicians in China. Nonetheless, over two-thirds of BHDS families were managed in Beijing. Genetic testing is centralized to certain tertiary referral centers in China and there is a national rare lung disease referral center (Peking Union Medical College Hospital) that might lead to such bias. This huge imbalance of diagnosis and management on patients with rare diseases between Beijing and the rest of China suggests urgent need for improvements in the recognition of rare diseases nation-wide.

As in prior reports, respiratory system was the most frequently affected in Chinese patients with BHDS with 92.4% of our cohort manifesting lung cysts (Table 3). A study of 312 Japanese BHDS patients reported that almost all patients had lung cysts [7]. Similarly, a study of Korean BHDS which enrolled 12 patients reported all cases had lung cysts [29]. On the contrary, it was reported that the prevalence of lung cysts of BHDS in USA was 85%, which was lower compared to the Japanese cohort [7, 30]. This might indicate that Asian patients were more prone to pulmonary cysts than Caucasian patients who account for 94% of patients in USA cohorts [30]. However, most of cases with BHDS in our study was diagnosed by respiratory physicians which may have introduced a bias. The widespread use of high-resolution computed tomography in recent decades has provided better detection of cystic lung diseases. As a result, cystic lung diseases such as BHDS have been better recognized and distinguished from emphysema. In addition, Fabre et al. reported a retrospective analysis of imaging and lung tissue resected during surgical management of pneumothorax in BHD cases, and matched this cohort with cases of spontaneous pneumothorax occurring in non-BHD patients [31]. BHD lungs showed punch-out cysts in the lower lobes with no inflammation, and lacked subpleural fibroelastotic scars and smoking changes different from non-BHD lungs, thus, recurrent pneumothorax in a non-smoker, in particular in a woman should be alerted [31, 32].

Due to the high prevalence of lung cysts in adult patients with BHDS, spontaneous pneumothorax has been found to be a common presentation [3]. In our study, 71% of confirmed patients had at least one episode of pneumothorax and nearly 85% of them had a family history of pneumothorax. This was consistent with the features of patients with BHDS reported from other Asian countries [10]. A recent survey of 104 American patients with BHDS reported a similar rate of spontaneous pneumothorax during their lifetime [30]. BHDS-related pneumothorax seems to have a higher recurrence rate after conservative therapy (observation, chest drainage and chemical pleurodesis) compared to surgical management. Liu et al. reported only 9% of patients had recurrence of pneumothorax after surgical treatment (pulmonary bullectomy and/or pleurodesis) while 52% of recurrence rate was observed after conservative treatment [25]. Gupta et al. reported pleurodesis (chemical or surgical) to reduce the ipsilateral recurrence rate by half (33% versus 63%) [30]. Another diffuse cystic lung disease LAM is also associated with high recurrence rate of pneumothorax. In 2017, American Thoracic Society/Japanese Respiratory Society Guideline suggested that patients with LAM be offered ipsilateral pleurodesis after their initial pneumothorax instead of waiting for the recurrence of the pneumothorax [33].

Renal cell cancer has been found to be the main manifestation associated with the poor prognosis of BHDS. Zbar et al. found a sevenfold increase in the risk of renal tumors for BHD-affected family members when adjusted for age [34]. Subsequent study revealed that the prevalence of renal tumors in patients with BHD was 34% with a mean age at diagnosis of approximately 50 years [35]. Chromophobe renal carcinoma was the most common histological type of renal cancer, followed by hybrid oncocytic/chromophobe tumor [3, 7]. Similar findings were reported in a recent largest study including 312 Japanese cases from 120 different families [7]. Renal carcinoma was detected in 8 (3.6%) patients in this study while that in Japan was 34.8% and 25% in Korea, respectively [7, 29]. The differences between these countries might attribute to less awareness and evaluation for renal manifestations of BHDS among Chinese physicians or ethnic differences; further studies are needed on this issue. In addition, lack of follow-up evaluations might have contributed to the low prevalence of renal cancer in the Chinese cohort. Thus, a long-term prospective observation study is needed to explore the true incidence of renal tumors among BHDS-affected families in the Chinese population.

Skin lesions were a common feature of BHDS in Caucasians, in whom it is found in about 80–90% [3, 36]. Characteristic lesions fibrofolliculomas and trichodiscomas are important clues in recognizing BHDS, but the patients in Asian countries seem to have lower prevalence of skin lesions [3, 7, 29]. Previous study demonstrated skin lesions developed in less than 30% in Chinese patients [10], similar to the current study. Only 6 patients were histologically diagnosed to have fibrofolliculomas-the typical skin lesion of BHD syndrome. Cutaneous sarcoma is rare in BHDS patients. In 2008, dermatofibrosarcoma protuberans and cutaneous leiomyosarcoma were firstly reported in American BHD families but determining whether they are part of the clinical spectrum of BHDS remains to be investigated in further studies [35]. However, this probably represents an underestimation due to limited diagnostic capacity of this rare disease, especially among general pulmonologists. The incidence of skin lesions among patients with BHDS reported from Peking Union Medical College Hospital, a national referral center for rare respiratory diseases, significantly increased from 11% (3/27) in 2017 to 47.2% (17/36) in 2019 [9, 22]. The awareness of characteristic skin manifestations was likely an important factor in the increased diagnosis rate over the interval. Similarly, dermatologist Chikako Iwabuchi and colleagues reported the increasing diagnosis rates of skin lesions in 31 Japanese individuals [37]. Skin lesions were recognized in over 80% of BHDS patients, including almost 75% of them histologically confirmed by skin biopsies [37]. Therefore, skin lesions appear to be more prevalent than previously reported in East Asian population. Assessment of suspicious skin findings, including dermoscopy and skin biopsy, should be considered in the context of an experienced multidisciplinary team.

The *FLCN* mutations in exon 11 were found to be the most frequently detected among Caucasian population, mainly c.1285dup/del and c.1300G > C [38]. Our study also showed that the most frequent mutation in Chinese BHD patients was the single deletion, duplication of cytosine in codon 1285 of exon 11. Until now, no genetic clusters associated with regional variations has been reported in China. The correlation between genotype and phenotype has been explored in the past decades. There was rather high incidence of pneumothorax associate with *FLCN* hotspot mutation c.1285dup (60% risk) in this cohort. However, a cohort from Germany reported less than 50% of 197 patients with BHDS had pneumothorax. Significantly increased pneumothorax risks were observed for mutations c.1300G > C (59%) and c.250-2A > G (77%) compared with *FLCN* hotspot mutation c.1285dup (37% risk) [38]. We are not able to explain the discrepant rates of pneumothorax associated with the mutation c.1285dup in Chinese patients compared to the Germany cohort. It would need further research on large-scale population to explore the correlation between these mutation and phenotype. Genetic testing methods of *FLCN* are of great significance for the diagnosis of BHD syndrome. Sanger sequencing was the most common method for diagnosing BHD. However, for those whose Sanger sequencing showed negative mutation results, a multiplex ligation dependent-probe amplification (MLPA) test was applied. MLPA can be used to detect whole-exon deletions and duplications that are not detectable by traditional Sanger sequencing [22]. Furthermore, when a large number of candidate genes are to be screened or there is no clue about responsible genes, whole-exome sequencing can make big differences [13].

To date, there is no effective treatment for BHDS, but the benefits of an early diagnosis include appropriate screening for renal tumors among BHDS individuals, patient education and family counseling. Another benefit of timely diagnosis is to provide appropriate treatment for BHDS patients with pneumothorax, as it is possible to reduce pneumothorax recurrence through surgical treatment. An average delay from first symptom to diagnosis was up to 13 years in 104 patients reported from USA [30]. The misdiagnosis was quite common. Previous results have demonstrated lower prevalence of skin lesions and renal tumors among of BHDS patients in China [9, 10]. Thus, the presenting manifestation of pneumothorax associated with characteristic lung cysts are important clues to achieve an early diagnosis. A study in Japan established a scoring system that has a high degree of sensitivity and specificity for distinguishing suspected BHDS from primary spontaneous pneumothorax [39]. Several radiological studies also found appropriate evaluation of chest CT images to be helpful in differentiating BHDS from other diffuse cystic lung diseases [40–44]. Therefore, the most common presentation pneumothorax in setting of characteristic cystic lung lesions or a family history of pneumothorax should suggest the need for *FLCN* gene testing to make timely diagnosis.

There were several limitations of this study. Firstly, this was a retrospective study, and majority of reported cases were from tertiary referral centers in Beijing. Secondly, some cases lacked data regarding the *FLCN* gene mutations as well as aspects of management and follow-up. However, this study collected all the consecutively cases reported in the Chinese population in recent decades and fully described the real-life diagnosis and managements of BHDS which should be valuable for better understanding this rare disease in the coming years.

## Conclusion

Although BHDS has been increasingly reported in the recent years in China, only minority of the cases were managed in the regions beyond Beijing and Jiangsu Province. The dominant clinical manifestation was pulmonary cysts associated with recurrent pneumothorax, while skin lesions and renal tumors were less reported. Lack of disease awareness, delayed diagnosis along with suboptimal management present critical challenges for Chinese BHDS patients.

## Data Availability

The datasets generated and analyzed for this study are not publicly available due to participant privacy but are available from the corresponding author upon reasonable request.
